# Antibacterial and Anti-Quorum Sensing Molecular Composition Derived from *Quercus cortex* (Oak bark) Extract

**DOI:** 10.3390/molecules200917093

**Published:** 2015-09-18

**Authors:** Dmitry G. Deryabin, Anna A. Tolmacheva

**Affiliations:** Microbiological Department, Orenburg State University, 13 Pobedy Avenue, Orenburg 460018, Russia; E-Mail: annatolmacheva56@gmail.com

**Keywords:** plant-derived molecules, antimicrobials, quorum sensing, *Quercus cortex*, *Chromobacterium violaceum*

## Abstract

*Quercus cortex* (Oak bark) has been used in European folk medicine since medieval times for treatment of diarrhea, stomatitis, pharyngitis and skin inflammations. Its antimicrobial activity is a well-known therapeutic property of oak bark, and its novel anti-quorum sensing (QS) ability has also been described recently. In this study, we examined the bioactive compounds of *Quercus cortex* extract and compared their direct antibacterial and regulatory anti-QS effects against *Chromobacterium violaceum* CV026 in a biotest. Evaluation of the original *Quercus cortex* extract showed weak antibacterial and prominent anti-QS activities that were retained and completely restored when the samples were dried and re-hydrated. The one-step liquid chromatography result indicated that the anti-QS activity might be determined by hydrophobic compounds; however, the subsequent reverse phase high performance liquid chromatography led to dissipation and loss of the activity. The gas chromatography–mass spectrometry gave excellent resolution between a majority of the compounds. Based on this result, 10 of the 35 identified small molecules were selected for further screening. The subsequent investigation indicated several compounds determined both the antibacterial and anti-QS activities of the *Quercus cortex* extract. Direct antibacterial activity was shown for 1,2,3-benzenetriol and 4-propyl-1,3-benzenediol, while sub-inhibitory concentrations of these compounds led to anti-QS effects. Five compounds: 4-(3-hydroxy-1-propenyl)-2-methoxy-phenol; 3,4,5-trimethoxyphenol; 4-hydroxy-3-methoxybenzaldehyde; 7-hydroxy-6-methoxy-2*H*-1-benzopyran-2-one and 2*H*-1-benzopyran-2-one were characterized as QS inhibitors independent of any effect on bacterial growth. Biologically relevant concentrations of each single component showed weak activity only while reconstruction of the small molecule composition derived from the *Quercus cortex* extract provided comparable complementary activity against *C. violaceum* CV026 in the biotest as the crude extract.

## 1. Introduction

The *Quercus* genus belongs to the family *Fagacae*, subfamily *Quercoideae*, and contains about 400 species widespread in Europe, Asia and America. Since medieval times, the bark of these trees has been used in traditional medicine and applied topically to burns and wounds, or applied orally for gastrointestinal diseases. In the modern European Pharmacopoeia [1], it is described as the cut and dried bark of young branches and the lateral shoots of *Quercus robur*, *Quercus petraea* and/or *Quercus pubescens* that are harvested in spring from March to April. The most common medical indications for *Quercus cortex* (Oak bark) extract include symptomatic treatment of mild diarrhoea, minor inflammation of the oral mucosa or skin. Oak bark powder from *Q. robur* is also used for prophylaxis of diarrhea in cattle, horses, pigs, sheep and chicken [2].

The well-known rationale for the therapeutic use of *Quercus cortex* is its direct antibacterial activity against many bacteria pathogenic for humans and animals. The antimicrobial effect of Mediterranean *Quercus ilex* bark extracts was previously revealed against *Brucella*, *Enterobacter*, *Escherichia*, *Neisseria*, *Pseudomonas* and *Bacillus*, while quite potent antibacterial effects have also been shown against *Escherichia coli* strains [3]. In the following articles, the expressed activity of *Quercus infectoria* ethanolic extracts was suggested against the clinically relevant entero-haemorrhagic *E. coli* 0157:H7 strain [4,5]. The antimicrobial activity of *Q. robur* bark extracts prepared using a stepwise-gradient separation was described against *Staphylococcus aureus* (*S. aureus*), *Enterobacter aerogenes* and *Candida albicans*, as well as dependence of this bioactivity from extractant polarity was shown [6]. Evident antibacterial activity against hospital methicillin-resistant *S. aureus* isolates was also reported for aqueous and ethanolic extracts of *Q. infectoria* that might provide a new phytopharmaceutical treatment effective against antibiotic-resistant bacterial pathogens [7].

Current understanding of medicinal plants’ anti-infectious potential includes their impact on the cell-to-cell communication mechanism in bacteria denoted as “quorum sensing” (QS). Typical QS involves the generation and release of small diffusible signal molecules—autoinducers; they accumulate in the environment to a certain threshold concentration, followed by recognition by receptor proteins that regulate the expression of a particular set of genes and control manifold activities [8]. Since this mechanism is responsible for bacterial virulence induction, QS targeting could be a promising strategy to control pathogenic bacteria [9], and some medicinal plants are capable of inhibiting QS-related processes [10]. An anti-QS property was previously described in *Quercus virginiana* [11], and this activity was confirmed in commercially available ethno-pharmacological *Quercus cortex* products [12].

Oak bark is usually described as a source of polyphenolic secondary metabolites: hydrolysable tannins, previously known as pyrogallol tannins, and condensed tannins—proanthocyanidins [13,14]. The total content of tannins in the dried substance varies from three to 20 percent, depending on the time of harvesting, the age of the branches and the method of assay used. Their antimicrobial effect is attributed to these tannins [15,16], possibly via inactivation of microbial adhesins, enzymes and cell envelope transport proteins. In turn, the tannins’ major mechanism of action is considered their ability to hydrogen bond their available polyhydroxyphenolic groups with proteins (especially proline-rich proteins) so the multivalent cross-linking leads to precipitation and aggregation effects [17,18]. In this context, it is very interesting that certain hydrolysable tannins (ellagitannins), namely vescalagin and castalagin, first isolated from a medicinal plant of Southern Florida, *Conocarpus erectus*, exhibited anti-QS properties [19].

In this study, we examined the bioactive molecules of *Quercus cortex* (Oak bark) extract and compared their direct antibacterial and regulatory anti-QS effects against *Chromobacterium violaceum* (*C. violaceum*) CV026 in a biotest.

## 2. Results and Discussion

### 2.1. The Effects of the Total Quercus cortex Extract in an Agar-Diffusion Assay

The commercially available ethnopharmacological product *Quercus cortex* was used for the primary sample “A” preparation: 20 g of plant material was ground to a fine powder, added to 200 mL of hot sterile distilled water and boiled for 30 min. The cooled aqueous extract was centrifuged to remove particulate matter and the supernatant was filtered through a 0.2 μm polyethersulfone syringe filter to ensure the sterility of the “A” sample. Part of this sample was then dried in an oven at a temperature of 60 °C and the obtained sample “B” was collected and weighed; according to this procedure, the total extractable dry matter from the oak bark was 10%. The desalted sample “C” was prepared from sample “B” by one-step liquid chromatography on a Synchroprep RP-P C8 phase column (SynChrom Inc., Lafayette, IN, USA). The total bonded fraction was eluted, evaporated in a vacuum centrifuge and lyophilized. Prior to the bioactivity assays, the dry samples were re-dissolved in sterile distilled water to a concentration equivalent to the primary “A” sample (see Section 3.1), and all the samples were screened in a semi-quantitative agar-diffusion assay against *C. violaceum* CV026 to evaluate the antibacterial and anti-QS activities of the total *Quercus cortex* extract.

**Figure 1:**
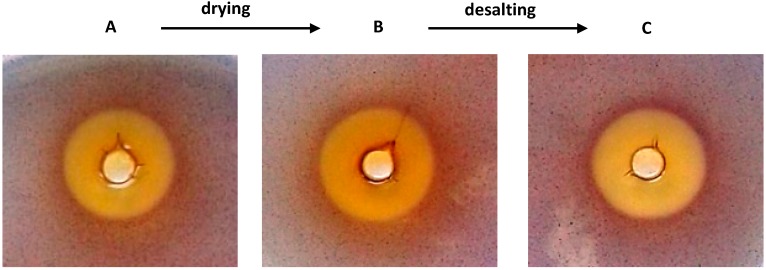
Comparison of antibacterial and anti-QS properties in the “A”, “B” and “C” samples derived from *Quercus cortex* evaluated with a semi-quantitative agar-diffusion assay.

Only weak antibacterial activity was observed in the “A” and “B” samples that led to *C. violaceum* CV026 growth inhibition zones in the wells of 33 ± 4 mm^2^ and 28 ± 3 mm^2^, respectively (Figure 1). On the other hand, the diffusion test results showed prominent anti-QS activity manifested in visible halos of 175 ± 16 mm^2^ (sample “A”), 178 ± 16 mm^2^ (sample “B”) and 171 ± 13 mm^2^ (sample “C”).

These data showed QS regulatory activities in the *Quercus cortex* extract were more notable than direct antibacterial activity, in good agreement with earlier results [11,12]. Significantly, the extract retained these bioactivities when it was dried and they were completely restored upon re-hydration as determined by a comparison of the “A” and “B” samples. Another important fact is the preliminary determination that the anti-QS activity resides in hydrophobic compounds, as the “C” fraction eluted during one-step liquid chromatography on a reversed-phase column retained 96% of the bioactivity of the original plant extract. Thus, derived from the “A” sample, dried “B”, followed by desalted “C” samples were prepared for the following chromatographic analysis and retained their bioactivity when evaluated with the *C. violaceum* CV026 assay.

### 2.2. Reverse Phase High Performance Liquid Chromatography (RP-HPLC) Analysis of Quercus cortex Extract and Screening of Fractions

The desalted “C” fraction of the *Quercus cortex* extract was separated by the RP-HPLC method in linear acetonitrile gradients, using parameters similar to those described previously for separation of tannin-related anti-QS compounds in *Conocarpus erectus* extract [20]. With baseline absorbance at 313 nm this procedure yielded 16 fractions that were evaporated, lyophilized and analyzed for antibacterial and anti-QS properties with a semi-quantitative agar diffusion assay.

In contrast to the data provided by Adonizio *et al*. [20], our results showed some limitations of RP-HPLC as follows: (1) there was no clear separation of *Quercus cortex* compounds because most fractions were eluted over a narrow range of acetonitrile concentrations (28% to 36%); (2) the identification of each fraction was based on a combination of retention time and spectral matching and they had multiple components; and (3) there is no accumulation of antibacterial or anti-QS activity in any fraction. Moreover, we observed the dissipation and loss of activities that were unstable and weakly detected in certain fractions collected in triplicate assays.

Thus, against expectations, the reverse phase high performance liquid chromatography did not reveal ellagitannins or other components similar to *C. erectus* anti-QS compounds [19], probably because of the distinctive nature of this phenomenon in the *Quercus cortex* extract. This negative result required the use of another methodological approach, *i.e.*, gas chromatography-mass spectrometry analysis.

### 2.3. Gas Chromatography-Mass Spectrometry (GC-MS) Analysis of Quercus cortex Extract and Compound Library

The dried “B” sample of the total *Quercus cortex* extract was re-solved in methanol and directly analyzed by the GC-MS method that is currently used to search the small antimicrobial plant-derived molecules [21].

**Figure 2:**
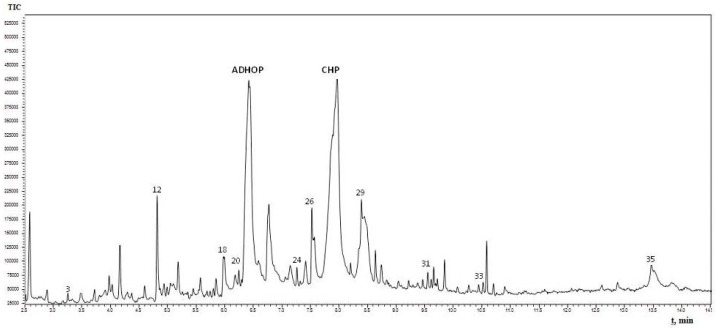
GC-MS chromatogram of *Quercus cortex* extract (“B” sample). Where: (ADHOP) 2-amino-9-[3,4-dihydroxy-5-(hydroxymethyl)oxolan-2-yl]-3*H*-purin-6-one, (CHP) cyclohexane-1,2,3,4,5-pentol, (**3**) furan-2-carboxylic acid, (**12**) 5-(hydroxymethyl)-2-furaldehyde, (**18**) 1,2,3-benzenetriol, (**20**) 4-hydroxy-3-methoxybenzaldehyde, (**24**) 4-hydroxy-3-methoxybenzoic acid, (**26**) 4-propyl-1,3-benzenediol, (**29**) 4-(3-hydroxy-1-propenyl)-2-methoxy-phenol, (**31**) 7-hydroxy-6-methoxy-2*H*-1-benzopyran-2-one, (**33**) 2*H*-1-benzopyran-2-one, (**35**) 3,4,5-trimethoxy-phenol.

The results of the GC-MS chromatogram (Figure 2) showed excellent resolution between a majority of the compounds of interest. The 35 components detected with retention times in the range from 2.5 to 14.0 min presented in Table 1.

**Table 1:** The phytochemicals identified in the *Quercus cortex* extract by gas chromatography-mass spectrometry ^#^.

No.	Name of the Identified Compounds (IUPAC)	Retention Time, min	Peak Area, %
1	propane-1,2,3-triol *	2.590	3.56
2	decane *	2.885	0.30
3	furan-2-carboxylic acid *	3.250	0.30
4	1,3,5-triazine-2,4,6-triamine *	3.500	0.59
5	pentadecane *	3.755	0.25
6	2,3-dihydroxypropanal *	3.995	0.35
7	butanedioic acid *	4.015	0.30
8	2,3-dihydro-3,5-dihydroxy-6-methyl-4*H*-pyran-4-one *	4.170	1.19
9	2-amino-9-[3,4-dihydroxy-5-(hydroxymethyl)oxolan-2-yl]-3*H*-purin-6-one *	4.270	0.59
10	cyclopentane-1,2-diol **	4.385	0.30
11	1,2: 5,6-dianhydrogalactitol **	4.695	0.89
12	5-(hydroxymethyl)-2-furaldehyde *	4.825	1.98
13	(*R*)-2-acetamido-3-sulfanylpropanoic acid *	4.955	0.89
14	2-propenoic acid, 1-methylundecyl ester **	4.995	1.39
15	2,3-dihydro-3,5-dihydroxy-6-methyl-4*H*-pyran-4-one **	5.175	0.79
16	1-(2-hydroxyethyl)-4-methylpiperazine **	5.750	1.33
17	6-(4-hydroxy-6-methoxy-2-methyl-tetrahydro-pyran-3-yloxy)-2-methyl-dihydro-pyran-3-one **	5.890	0.79
18	1,2,3-benzenetriol *	5.965	0.99
19	2-methyl-5-nitro-pyrimidine-4,6-diol **	5.975	0.99
20	4-hydroxy-3-methoxybenzaldehyde *	6.260	0.53
21	2-amino-9-[3,4-dihydroxy-5- (hydroxymethyl)oxolan-2-yl]-3*H*-purin-6-one *	6.445	25.6
22	1,6-anhydro-β-d-glucopyranose *	6.775	6.14
23	1- (β-d-arabinofuranosyl)-4-*O*-trifluoromethyl uracil **	7.185	0.99
24	4-hydroxy-3-methoxybenzoic acid **	7.255	0.69
25	1,6-anhydro-β-d-glucofuranose *	7.400	0.89
26	4-propyl-1,3-benzenediol *	7.525	1.38
27	cyclohexane-1,2,3,4,5-pentol *	7.970	36.38
28	4-(hydroxymethyl)-2,6-dimethoxyphenol *	8.225	0.37
29	4-(3-hydroxy-1-propenyl)-2-methoxy-phenol *	8.405	4.45
30	9-[(2*R*,3*R*,4*S*,5*R*)-3,4-dihydroxy-5-(hydroxymethyl)oxolan-2-yl]-3*H*-purine-2,6-dione *	9.465	0.30
31	7-hydroxy-6-methoxy-2*H*-1-benzopyran-2-one *	9.545	0.48
32	methyl-α-d-glucopyranoside *	9.835	1.19
33	2*H*-1-benzopyran-2-one *	10.505	0.30
34	2-ethoxy-6-(methoxymethyl)phenol **	10.935	0.75
35	3,4,5-trimethoxy-phenol **	13.490	1.79

^#^ Compounds identified by direct comparison with authentic reference samples according NIST, Mainlib, CAS, Wiley9, and DD2012 libraries. * identified compounds to have a match in the library of greater than 90%. ** identified compounds to have a match in the library less than 90%.

There were two components in high abundance that were detected with retention times of 6.445 and 7.970 min, and were identified as purine nucleoside 2-amino-9-[3,4-dihydroxy-5-(hydroxymethyl)oxolan-2-yl]-3*H*-purin-6-one (25.6% peak area) and carbohydrate cyclohexane-1,2,3,4,5-pentol (36.38% peak area). The first compound has been previously described as cytotoxic against B-lymphoblast and T-cell lines [22] as well as an antiviral agent against Vero cells infected with HSV-1 [23]. The second compound and its inositiol-related derivatives are common in biochemical pathways of plant-associated alpha-proteobacteria, where the influence of inositol catabolism on microbe-plant interactions has recently been described [24], however, it is unlikely that this simple sugar metabolite to have a role in QS inhibition, either. Some other linear molecules (glycerol, butanedioic acid, propanal, glycerol acetate, *etc.*) and sugars (1,6-anhydro-beta-d-glycopyranose; 1,6-anhydro-beta-d-glucofuranose; methyl-alpha-d-glucopyranoside, *etc.*) were simple metabolites too with no signs of possible effects on cell-to-cell communication systems.

On the other hand, based on the criteria: (i) an amphiphilic structure that offers the possibility of trans-membrane migration with targeting of the QS system; (ii) at least 0.3% of the peak area; and (iii) literature data about bacteria-related activities, 10 compounds identified by GC-MS in the *Quercus cortex* extract were selected for further screening. The molecular structure of these compounds is shown in Table 2, where the list of compounds includes furan-2-carboxylic acid (0.3%); 5-(hydroxyl-methyl)-2-furaldehyde (1.98%); 1,2,3-benzenetriol (0.99%); 4-hydroxy-3-methoxy-benzaldehyde (0.53%); 4-hydroxy-3-methoxybenzoic acid (0.69%); 4-propyl-1,3-benzenediol (1.38%), (**7**) 4-(3-hydroxy-1-propenyl)-2-methoxy-phenol (4.45%); 7-hydroxy-6-methoxy-2*H*-1-benzopyran-2-one (0.48%); 2*H*-1-benzopyran-2-one (0.3%) and 3,4,5-trimethoxy-phenol (1.79%). A library of these chemically synthesized compounds, which were completely identical in structure to the compounds identified in the oak bark extract, was designed and submitted to the growth and violacein quantification assay.

### 2.4. Screening the Library of Compounds in the Growth and Violacein Quantification Assay

**Figure 3:**
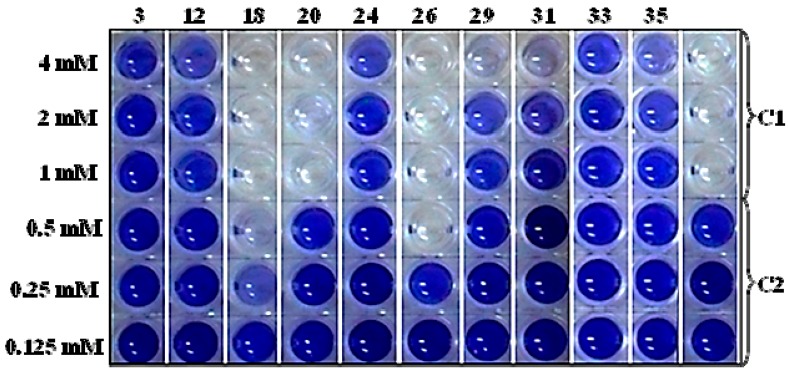
Anti-QS effects of selected compounds evaluated for violacein inhibition in a *C. violaceum* CV026 quantification assay. Columns: (**3**) furan-2-carboxylic acid, (**12**) 5-(hydroxymethyl)-2-furaldehyde, (**18**) 1,2,3-benzenetriol, (**20**) 4-hydroxy-3-methoxybenzaldehyde, (**24**) 4-hydroxy-3-methoxybenzoic acid, (**26**) 4-propyl-1,3-benzenediol, (**29**) 4-(3-hydroxy-1-propenyl)-2-methoxy-phenol, (**31**) 7-hydroxy-6-methoxy-2*H*-1-benzopyran-2-one, (**33**) 2*H*-1-benzopyran-2-one, (**35**) 3,4,5-trimethoxy-phenol; (**C1**) vehicle control without C6-AHL supplementation; (**C2**) positive control supplemented with C6-AHL, 10^−8^ mol/L. The concentrations of the compounds decreased in two-fold dilutions from the top row (4 mM) to the bottom row (0.125 mM).

The 10 selected compounds identical with small molecules derived from the *Q. cortex* extract were qualitatively analyzed in the bacterial growth inhibition and violacein production assay (Figure 3), and the results are presented in Table 2.

**Table 2:** Bioactivity of selected compounds presented as minimal inhibitory concentration (MIC) and effective concentration (EC_50_) that inhibited 50% of violacein production in a *C. violaceum* CV026 assay.

No.	Name of Compound (IUPAC); PubChem CID	Structural Formula	Bioactivity (mM)	
			MIC	EC_50_
3	furan-2-carboxylic acid; 6919	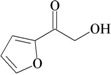	-	-
12	5-(hydroxymethyl)-2-furaldehyde; 237332	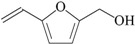	-	-
18	1,2,3-benzenetriol; 1057	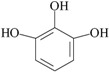	1.7	0.175
20	4-hydroxy-3-methoxybenzaldehyde; 1183	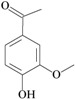	-	0.575
24	4-hydroxy-3-methoxybenzoic acid; 8468	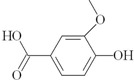	-	-
26	4-propyl-1,3-benzenediol; 87874	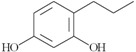	0.5	0.275
29	4- (3-hydroxy-1-propenyl) -2-methoxy- phenol; 1549095	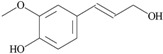	-	0.79
31	7-hydroxy-6-methoxy-2*H*-1-benzopyran-2-one; 5280460	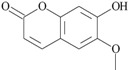	-	1.13
33	2*H*-1-benzopyran-2-one; 323	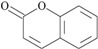	-	2.67
35	3,4,5-trimethoxy- phenol; 69505	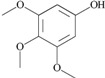	-	1
C_1_	tetracycline	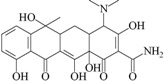	0.007	NT
C_2_	benzo[1,3]dioxol-5-yl amide hexanoic acid	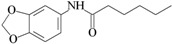	NT	0.052

C_1_—positive control (antibacterial); C_2_—positive control (anti-QS); NT—not tested.

Direct antibacterial activity detected as *C. violaceum* CV026 growth inhibition by measuring at 450 nm was shown for two compounds: 1,2,3-benzenetriol (MIC = 1.7 mM) and 4-propyl-1,3-benzenediol (MIC = 0.5 mM). On the other hand, sub-inhibitory concentrations of these compounds led to anti-QS effects presented as effective concentrations (EC_50_), which inhibited violacein production by 50%, calculated between the optical density (OD_575_) values in the C6-AHL free and C6-AHL containing control probes. The EC_50_ values of these compounds were 0.175 mM and 0.275 mM, respectively.

The 1,2,3-trihydroxybenzene (pyrogallol) belongs to phenolic compounds synthesized by the shikimate pathway in many plants and its presence is typical for *Quercus cortex,* which contains a minimum 3.0% tannins, expressed as pyrogallol. More recently, pyrogallol antibacterial activity has been described against many bacteria, including *Pseudomonas* spp. [25] and *Vibrio* spp. [26], and sub-inhibitory concentrations have been reported to inhibit QS-regulated bioluminescence in *Vibrio harveyi* [27]. The 4-propyl-1,3-benzenediol is a phenolic (resorcinolic) lipid common in plants, where their ability to inhibit bacterial growth [28] and antagonize bacterial QS [29] has been shown previously. Thus, in the present study, we confirm these data and report for the first time 1,2,3-trihydroxybenzene and 4-propyl-1,3-benzenediol are important *Quercus cortex* compounds responsible for both antibacterial and anti-QS activities.

Some of the other compounds did not cause any observable *C. violaceum* CV026 growth inhibition, at the same time showing varying degrees of anti-QS effect.

The most pronounced activity (EC_50_ = 0.575 mM) was shown for 4-hydroxy-3-methoxybenzaldehyde (vanillin), first described as a QS inhibitor in *Vanilla planifolia* bean extract [30] and subsequently demonstrated significant inhibition in short-chain and long-chain AHL-induced biotests [31]. In contrast, the structurally similar compound 4-hydroxy-3-methoxy-benzoic (vanillic) acid does not possess this bioactivity.

The 4-(3-hydroxy-1-propenyl)-2-methoxy-phenol benzenediol (coniferyl alcohol), one of the main phenolic compounds in *Quercus cortex* extracts, showed moderate violacein inhibition with an EC_50_ = 0.8 mM. Previously this coniferyl alcohol was described as a natural inducer of *nod* genes in certain rhizobial species [32] and now we are the first to describe this plant-derived small molecule as a QS inhibitor. In this context, interestingly, the coniferyl alcohol is structurally similar to eugenol, for which an anti-QS effect at subinhibitory concentrations has recently been shown [33].

Another previously undescribed phenolic compound with a moderate anti-QS activity (EC_50_ = 1 mM) was 3,4,5-trimethoxyphenol (antiarol). The same compound was previously isolated from an extract of the stem bark of the African tree *Antiaris africana* [34] as antiarol cinnamate; however, evidence of any activity against bacteria is absent.

Two structurally similar compounds: 2*H*-1-benzopyran-2-one (coumarin) and its derivative 7-hydroxy-6-methoxy-2*H*-1-benzopyran-2-one (scopoletin), belonging to the benzopyrone chemical class, showed EC_50_ values of 2.67 mM and 1.13 mM, respectively. Recently, coumarin has been described as a novel plant-derived QS inhibitor active against short, medium and long chain AHL, independent of any effect on bacterial growth [35]. In the present study, we confirmed the activity of coumarin and extend this view to scopoletin, indicating their similarities with naturally occurring furocoumarins from grapefruit, which have shown inhibition of QS signalling and QS-dependent biofilm formation in bacteria [36].

At the same time, two other structurally similar compounds: 2-furancarboxylic and 5-(hydroxymethyl)-2-furancarboxaldehyde, previously identified as factors that inhibit bacterial swarming and extracellular polysaccharide production by several bacteria [37], did not show observable activity in the *C. violaceum* CV026 growth and violacein production assay.

Thus, screening of a library of compounds designed according to the GC-MS results identified several phytochemicals that contribute to the antibacterial and anti-QS activities of the *Quercus cortex* extract. Seven of the ten tested compounds exhibited the presence of these activities to varying degrees, some of which confirmed the available data and some of which have been described for the first time. 

### 2.5. Reconstruction of Molecular Composition Derived from Quercus cortex Extract

**Figure 4:**
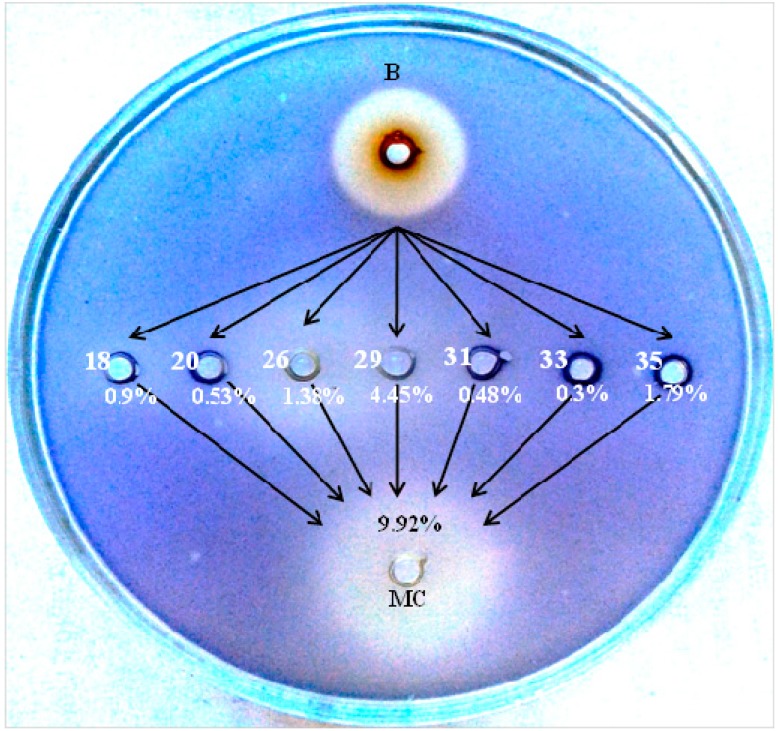
Comparison of bioactivity of original *Quercus cortex* “B” sample, relevant concentrations of selected chemical compounds identified in the *Quercus cortex* extract by GC-MS, and a reconstructed *Quercus cortex* extract, evaluated with a semi-quantitative agar-diffusion assay. Where: “B” sample, (18, 20, 26, 29, 31, 33, 35) selected compounds whose designations are identical to those in Table 2, (MC) molecular composition.

A comparison of the active compounds from *Quercus cortex* extract with tetracycline showed each of them was less active than the control antibiotic (MIC = 0.007 mM), and a comparison with benzo[1,3]dioxol-5-yl amide hexanoic acid showed they were less active than the AHL-mimetic compound (EC_50_ = 0.052 mM) used as an anti-QS positive control. In addition, biologically relevant concentrations of each component evaluated in an agar diffusion assay showed only weak activity (Figure 4), similar to the effects of some of the fractions collected with reverse phase high performance liquid chromatography. This may explain the dissipation of bioactivities after the RP-HPLC procedure, which may have occurred because the active compounds were separated over numerous fractions. Thus, in contrast to previously reported data and following the paradigm “one plant → one active compound → one biological effect” [11,30,34], the obtained result did not show any major antibacterial or anti-QS compound in *Quercus cortex* extract.

This result triggered the reconstruction of the original small molecule composition consisting of seven active plant-derived compounds taking into account their quantitative content evaluated as a peak area in the GC-MS analysis. The formula of this “molecular cocktail” included 4-(3-hydroxy-1-propenyl)-2-methoxy-phenol (0.445 mg/mL); 3,4,5-trimethoxyphenol (0.179 mg/mL); 4-propyl-1,3-benzenediol (0.138 mg/mL); 1,2,3-benzenetriol (0.099 mg/mL); for 4-hydroxy-3-methoxybenzaldehyde (0.053 mg/mL); 7-hydroxy-6-methoxy-2*H*-1-benzopyran-2-one (0.048 mg/mL) and 2*H*-benzopyran-2-one (0.030 mg/mL), which in sum represented 9.92% of the total peak area of volatile constituents detected in the GC-MS chromatogram, and approximately corresponded to the amount of these compounds in the total dry matter extractable from the oak bark biomass.

Subsequent evaluation of the antibacterial and anti-QS activity of the molecular reconstruction in the semi-quantitative agar diffusion assay showed it not only reproduced but even exhibited superior bioactivity in comparison with the initial *Quercus cortex* extract (Figure 4). The violacein inhibition halo zone was 235 ± 19 mm^2^; 132% of the anti-QS activity in sample “B” without any observable changes in growth inhibition zones.

Thus, the obtained result presents the bioactivity of *Quercus cortex* extract as a manifestation of a complementary poly-component composition consisting of seven small plant-derived molecules without single any lead compound. It is not clear whether the composition is in the living plant or is the result of post-fermentation and hydrolysis processes in the collected raw material; however, we think that the therapeutic effect of *Quercus cortex* extract on bacterial infections is precisely determined by the bioactivity of this molecular mixture.

## 3. Experimental Section

### 3.1. Plant Material and Extract Preparation

*Quercus robur L*. cortex was collected in southwest Russia in March 2011. It was purchased as a cut and dried oak bark ethnopharmaceutical product (batch number 010311 Ф8) from Phytopharm Ltd. (Anapa, Krasnodar region, Russia). A voucher specimen (ICIS 001302) was deposited in the regional plant and bacterial samples collection at the Institute of Cellular and Intracellular Symbiosis of the Russian Academy of Sciences, Orenburg, Russia.

The plant material was ground to a fine powder using an electric mill and, according to the manufacturer’s recommendations, 20 g was added to 200 mL of hot sterile distilled water and boiled for 30 min. The cooled aqueous extract was centrifuged at 1000× *g* for 10 min to remove particulate matter and the supernatant was filtered through a 0.2 μm polyethersulfone syringe with a sterile filter (Membrane Solutions LLC, Lane Plano, TX, USA) into autoclaved vials. The 100 mL of this sample was then dried at a temperature of 60 °C and the obtained sample “B” was collected and weighed; the yield of dry extract was 10% from plant material used for primary sample “A” preparation. Prior to the bioactivity assays, sample “B” was re-dissolved in sterile distilled water to a concentration of 10 mg/mL that was equivalent to dry matter extractable from the primary “A” sample.

### 3.2. Reverse Phase High Performance Liquid Chromatography Procedure

The dry oak bark extract (5 mg of sample “B”) was dissolved in 25 mL of 0.1% trifluoroacetic acid (TFA) and was desalted by RP-HPLC on a Synchroprep RP-P C8 phase column (30 μm, dimensions 25 × 50 mm; SynChrom Inc., Lafayette, IN, USA). The total bonded fraction was eluted with 65% acetonitrile in water with 0.1% TFA. The obtained desalting sample “C” was evaporated in a vacuum centrifuge Speedvac (Savant, Midland, MI, USA) and lyophilized (Labonco, Kansas City, MO, USA).

The following RP-HPLC procedure was similar to that used for the determination of anti-QS compounds in *C. erectus* extract [20] with little modification. Briefly, sample “C” was dissolved in 0.1% TFA and separated on a Luna C18 column (5 µm, dimensions 4.6 × 150 mm; Phenomenex, Torrance, CA, USA) at ambient temperature. A water-acetonitrile mobile phase with 0.1% TFA was used with a flowrate of 1.5 mL/min. Conditions were as follows: 0% acetonitrile for 50 min, 36%–80% for 3 min, and isocratic 80% for 15 min. Fractions were collected manually based on absorbance at 313 nm, lyophilized and tested for antibacterial and anti-QS activity.

### 3.3. Gas Chromatography-Mass Spectrometry Analysis

The 10 mg of dry oak bark extract (sample “B”) was dissolved in 1 mL methanol with careful stirring and then used for GC-MS phyto-chemical analysis.

The GC-MS procedure was performed using a Shimadzu QCMS-QP2010 Plus system equipped with a HP-5MS column (length: 30.0 m, diameter: 0.25 mm, film thickness: 0.25 μm). The sample was introduced using a 10 μL Hamilton 1700 syringe. Helium gas (99.999%) was used as the carrier gas at a constant flow rate of 1 mL/min. The column oven temperature was 100 °C; the temperature was increased at a rate of 20 °C/min to 290 °C. The injector was set at 200 °C and the detector at 290 °C.

Software adopted to handle the mass spectra and chromatograms were GC-MS Solutions and GC-MS Postrun Analysis. Identification of compounds was carried out by comparing the retention times with those of authentic compounds and by matching spectra in reference National Institute of Standards and Technology (NIST), Mainlib, CAS, Wiley9, and DD2012 libraries. The relative percentage of each component was calculated by comparing its average peak area to the total area.

### 3.4. Compound Library

A chemical compound library for bioactivity screening was designing according to the GC-MS data. The selected laboratory grade chemicals, which were completely identical in structure to the compounds identified in the oak bark extract, were purchased from Sigma-Aldrich LTD and Enamine LTD. Prior to experiments these compounds were prepared fresh by dissolving in distilled water and sterilized by filtration using a 0.2 μm filter. The aliquots were diluted in sterile distilled water at concentrations ranging from biologically relevant to extremely high (0.1 M) in order to identify their full range of antibacterial and anti-QS effects.

### 3.5. Bacterial Strain and Culture Conditions

*Chromobacterium violaceum* NCTC 13274, originally described as CV026 [38], was acquired from the National Collection of Type Cultures. This strain was derived from wild-type *C. violaceum* ATCC 31532, which used a two-component QS system with the *cviI* gene responsible for cognate autoinducer *N*-hexanoyl-l-homoserine lactone (C6-AHL) synthesis, and *cviR* gene encoding a receptor protein that responds to C6-AHL and induces the characteristic expression of the purple pigment violacein. In turn, *C. violaceum* CV026 (HgR, *cvil*: Tn*5*, *xyl*E, KanR) is a colourless double mini-Tn5 mutant with an insertion in the *cvi*I gene, and is deficient in the production of its cognate autoinducer but retains responsiveness to exogenous short-chain AHLs manifested in violacein production. Anti-QS compounds inhibit this reaction, making *C. violaceum* CV026 excellent for screening both regulatory and direct antibacterial activities [11,12].

Bacterial strains were stored as 40% glycerol stocks at −20 °C and were cultured in Luria-Bertani (LB) broth medium supplemented with 30 μg/mL kanamycin at 27 °C.

C6-AHL was obtained from the Cayman Chemical Company LTD as a ≥95% pure crystalline solid. A 0.01 M stock solution of this QS exogenous inductor was prepared in 96% ethanol and kept at −20 °C before use.

### 3.6. Bacterial Growth Inhibition and Violacein Production Assays

The semi-quantitative analyses of direct antimicrobial and anti-QS activities in samples, fractions and chemical compounds were performed with an agar-diffusion assay as described previously [12]. Briefly, 5 mL of warm molten 0.5% LB agar was inoculated with 200 µL of freshly prepared *C. violaceum* CV026 culture OD_450_ = 0.5 U supplemented with C6-AHL (5 × 10^–8^ mol/L). The agar and inoculum were gently mixed and placed immediately over the Petri dish, which had been previously overlaid with a layer (10 mL) of 1.5% LB-agar. The solidified agar plates were punched with a flame-sterilized cork borer 5 mm in diameter and left undisturbed for 30 min for absorption of excess moisture. Then the wells were filled with 30 μL of appropriate samples, fractions or chemical compounds; the assay plates were incubated overnight at 27 °C for 24–48 h and were then examined for culture growth and violacein production. Direct antimicrobial activity was determined as the growth inhibition zone (mm^2^) around the wells and the anti-QS activity was detected as an area of pigment inhibition (mm^2^) *i.e.*, a colourless but viable halo on a purple background.

The growth and violacein quantification assay was performed using a Stat Fax 303 6VIS microstrip reader according to [29] with minimal revisions. Briefly, 20 μL of an overnight culture of *C. violaceum* CV026 was inoculated into 1 mL of LB broth containing C6-AHL (10^−8^ mol/L) and two-fold chemical compound dilutions from 4 mM to the 0.125 mM in the final sample. Sterile probes as well as inoculated probes with and without C6-AHL only were used as the controls. After 18–24 h of incubation at 27 °C, growth intensity was assessed by measuring the absorbance at 450 nm and the development of QS-dependent reactions was estimated by violacein production in the bacterial biomass harvested by centrifugation at 12,000 *g* for 5 min, extraction with 96% ethanol and quantification at 575 nm. The antibacterial activity was established as the minimal inhibitory concentrations (MIC), which completely excluded bacterial growth; the anti-QS effect was presented as EC_50_ concentrations which inhibited violacein production by 50%, calculated between the OD_575_ values in the C6-AHL free and C6-AHL containing control probes.

The controls used were: sterile distilled water as the vehicle control and *N*-hexanoyl-l-homoserine lactone (10^–^^8^ mol/L, ≥95%, Cayman Chemical Company, Ann Arbor, MI, USA) as the positive control. Antibiotic tetracycline (98%, Sigma-Aldrich, St. Louis, MO, USA) was also included for comparative purposes as the antibacterial compound active against the *C. violaceum* 026 strain [39]. The positive control for the quorum sensing inhibition assay was performed with benzo[1,3]dioxol-5-yl amide hexanoic acid (≥99%, Enamine LTD, Kiev, Ukraine), previously described as AHL-mimetic anti-QS compound [40].

### 3.7. Statistical Analysis

All assays were performed in triplicate and the significance of the data was tested using ANOVA (*p* < 0.05) employing the software package Statistica V8.

## 4. Conclusions

The antibacterial and anti-QS activities of *Quercus cortex* (Oak bark) extract were determined by a poly-component composition consisting of seven small plant-derived molecules without any single lead compound. This composition includes some phenolic compounds, four of which were described previously to possess anti-QS activity, and three were shown as QS inhibitors for the first time. The obtained result indicates the promise of novel synthetic poly-component drugs that mimic plant-derived molecular compositions without needless compounds to be active against bacterial pathogens that rely on QS signaling.

## Supplementary Materials

RP-HPLC chromatogram of the *Quercus cortex* extract (“C” sample) is available as Supplementary Materials. Supplementary Materials can be accessed at: http://www.mdpi.com/1420-3049/20/09/17093/s1.
