# Supplementary material for: Antibacterial and Anti-Quorum Sensing Molecular Composition Derived from Quercus cortex (Oak bark) Extract
Source: Molecules. 2015 Sep 18;20(9):17093–108. doi: 10.3390/molecules200917093 (PMC6332234; doi:10.3390/molecules200917093)
Supplement: Supplementary file 1 [file molecules-20-17093-s001.pdf]

Supplementary Materials

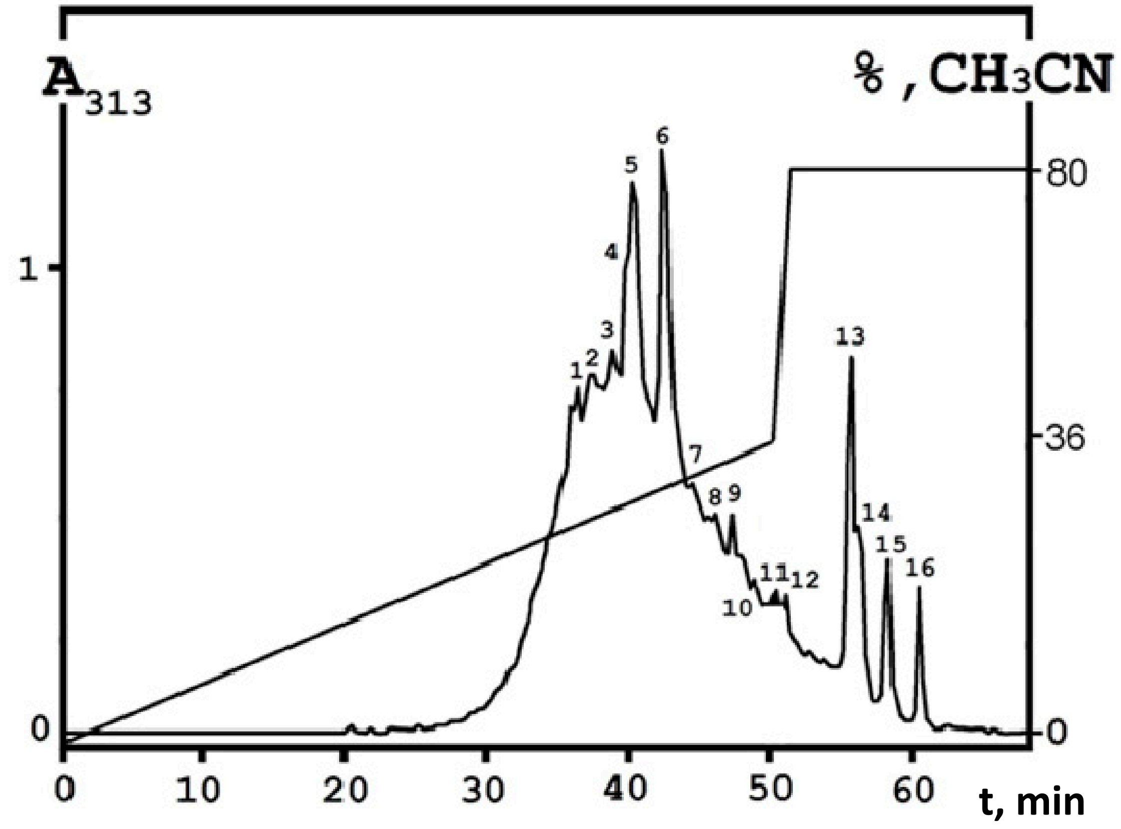

Figure S1. RP-HPLC chromatogram of the *Q. cortex* extract ("C" sample) obtained with Luna C_{18} column (Phenomenex, Torrance, CA, USA). Abscissa axis—retention time, min; ordinate axis—absorbance (A) at 313 nm. The acetonitrile (CH_3CN) concentration in the mobile phase presented as a linear gradient. Fractions 1–16 were collected, lyophilized and tested for antibacterial and anti-QS activity.
